# Multifaceted impacts of double-aging neighborhood’s built environments on SAIP: a deep dive into Chinese rapidly aging urban society

**DOI:** 10.3389/fpubh.2024.1504195

**Published:** 2025-01-15

**Authors:** Yue Qian, Guanmin Qiao, Guoping Zhang, Xianjing He, Renfeng Ma

**Affiliations:** ^1^Department of Geography and Spatial Information Techniques, Ningbo University, Ningbo, China; ^2^Donghai Academy, Ningbo University, Ningbo, China; ^3^Zhejiang Collaborative Innovation Center, Ningbo Universities Collaborative Innovation Center for Land and Marine Spatial Utilization and Governance Research, Ningbo University, Ningbo, China; ^4^Shenzhen Academy of Social Sciences, Shenzhen, China

**Keywords:** healthy aging, aging in place (AIP), successful aging in place (SAIP), built environment, home-based care

## Abstract

**Introduction:**

The healthy aging of older adults in dual-older adult communities is influenced by multiple factors, and understanding its underlying mechanisms can promote healthy aging among the older adults in a wide range of developing countries. This comprehensive study delves into the intricate interplay between multifaceted built environmental factors, and their direct and indirect effects on the successful AIP residing in double-aging neighborhoods.

**Methods:**

Applying a series of HLM, the research meticulously explores the intricate links between SAIP and multi-scale aging spaces, including home space, community social participation, and built environments.

**Results:**

The results show that: (1) Older adults people’s need for spiritual comfort derived from home space exceed the need for financial support and family care, becoming a major positive factor for SAIP; (2) The neighborhood based on acquaintance society, partly replace the role of home-based care in influencing SAIP. Especially, community participation has a positive impact, serving as an extension of the home space, such as college for senior citizens and outdoor activity space; (3) The built environment of double-aging neighborhoods has a significant positive effect, with a sense of place identity replacing the reliance on family members and acquaintances to facilitate SAIP; (4) In high-density old district, the distribution of public facilities is saturated, and the proper utilizes of these facilities becomes an important factor affecting SAIP.

**Discussion:**

We provide a multi-factorial perspective of SAIP, demonstrating the compensatory and substitutional roles of community-based older adults care services and friendly neighborhood relationships in fulfilling home-based older adults care functions. This approach better promotes the construction of age-friendly communities and supports SAIP.

## 1 Introduction

Since 2000, global aging has become an irreversible trend. The ubiquitous reality of “every family has elders, and everyone will grow old” underscores the phenomenon of population aging faced by all in today’s world. Developing countries are confronted with the challenge of aging before becoming affluent. Achieving successful aging has emerged as a crucial issue for human well-being. With the core family structure becoming more prevalent, the weakening of family caregiving functions and family capital (1), and the escalating demands of older adults for their later life (48), aging in place has become a popular social policy worldwide. This policy aims to provide older adults with the option to age in familiar surroundings while ensuring a certain level of independence and social support (2).

In the context of the unit community era, a large number of older adult Chinese people are concentrated in residential areas built during the 1950s to 1990s. These results in the simultaneous aging of both the built environment and residents within urban communities (3), a phenomenon referred to as “double aging.” The issues arising from building aging, such as high-density spaces, deteriorating structures, and a lack of aging-friendly facilities, pose threats to the AIP of the older adults. Meanwhile, the lack of demographic vitality due to population aging threatens urban development and renewal. Current researches often treat population aging and building aging as separate topics, failing to recognize their combined impact (4).

The complexity of the relationship among aging, health, and place goes far beyond government policy and societal perceptions (5). Lawton proposed the “P–E” framework to understand the nature of interactions between individuals and their living spaces (6). The P–E perspective attributes the aging experience of the older adults to their frequent interactions with the environment (7, 8), suggesting that successful aging experiences are the result of pursuing active health. This socio-economic issue of “dual aged” has significant implications for livability and resilience, yet viable solutions remain elusive (9).

Urban public spaces, as the most frequented venues for human interaction, cultivate a positive social atmosphere and social inclusivity that are significant factors in fostering a sense of place attachment (50). Thus, constructing new spaces that provide safety and healing for the older adults is a vital channel to promote proactive health among seniors in an aging society. The concept of home-based care emphasizes the location and source of socialized older adult care (10), focusing on professional older adults care facilities and supplies, but lacking attention to community spaces and built environments. With the participation of neighbors and residents, community spaces evolve into symbols of special significance and an essential foundation for choosing AIP, becoming vital spaces for AIP. Current research lacks an adequate understanding of the relationships between social organizational support, community reciprocity, social participation, and the preference for AIP (1, 11). Further investigations are needed to examine how and to what extent older adults social care support influences AIP. There is a lack of analysis on the interactions and correlations among different entities. The impact of the unique characteristics of acquaintance-based social mutual assistance and comprehensive infrastructure conditions (49) in China’s dual-older adult communities on local older adults care has become a topic of discussion.

Relying on the family for financial support, daily care, and emotional comfort has been the primary mode of older adults care in China. The sustainable functioning of this traditional model of older adults care relies on a relatively stable age structure of the population and a high fertility rate (12), and the primary differences in relevant research lie in the subject of responsibility or function (13–16). However, as China’s total fertility rate declines and the economy undergoes transformation, the capacity for home-based care is also evolving. Rapid urbanization has increased regional mobility, prompting children to whether actively or passively choose to work, study, and live away from their hometowns. This has impacted the traditional Chinese concept of older adults care, “When one’s parents are alive, one should not go on a long journey,” and family-based older adults care now faces multiple difficulties, including a lack of caregivers (12), inadequate provision of daily life care, and persistent neglect of emotional support (53). Research on home-based care in China has predominantly focused on analyzing self-care and family affordability amidst shifts in care models, relying heavily on empirical studies or theoretical discussions, with a disconnect between the concepts of home-based care and home-based care and a lack of comprehensive analysis at the family level (12). And little attention is paid to environmental factors in discussions of home-based care relationships.

Due to older adults’ familiarity with and identification with their environment (17, 18), as well as the inability of institutions such as nursing homes to provide an affordable and free lifestyle for ordinary people (19), AIP has become a universally preferred form of older adults care for the global older adults population in the 21st century. Furthermore, influenced by the culture of filial piety, Chinese older adult people highly value family cohesion and strong intergenerational connections (1), and hold the traditional belief of “returning to one’s roots.” As a result, AIP has also become the primary manifestation of older adults care in China. AIP is defined as “the ability to live safely, independently, and comfortably in one’s own home and community, regardless of age, income, or ability level” (20, 21). It emphasizes that the home and community space are the primary activity venues for older adults during the aging process and have diverse impacts on successful aging. Meanwhile, factors such as the location, space, size, and accessibility of the residence, as well as the natural environment’s temperature and air quality, and the artificial environment’s lighting, noise, and overall quality, all impact older adults’ sense of security and well-being in urban and housing environments, thereby influencing their health (22). Current research on AIP is mostly based on family-based older adult care (1, 12) and social older adult care (23, 24), emphasizing single-factor influence mechanisms while neglecting the combined effects of multiple factors.

Hence, incorporating the built environment, community-based and home-based older adults care into the SAIP system can effectively expand the theoretical connotation of local successful aging. In the context of the filial piety culture, exploring the impact of the built environment for dual-older adults households on SAIP can provide a basis for the revitalization of old cities. To study the multiple impacts of the built environment in dual-older adults communities on healthy aging, the core research questions are: (1) To what extent do home space and home-based care practices correlate with the SAIP, while controlling for individual socioeconomic conditions? (2) In the context of rapid urbanization, where home-based care support is increasingly compromised, how can community-based care support provide alternatives, compensation, and support for the SAIP of urban empty-nest seniors? (3) How do different dimensions of the built environment in double-aging neighborhoods affect the SAIP?

This article is composed of five chapters. Chapter 1 summarizes the current context of AIP in China, outlines the limitation of current researches, and poses three key questions about dual-older adult communities and successful aging. In chapter 2, we construct the framework of SAIP and introduce the research methodology by constructing the measurement of independent and dependent variables, outlining the study area, and describing the Hierarchical Linear Modeling (HLM) approach. Chapter 3 analyzes the results of the multi-level analysis, and Chapters 4 and 5 present the discussion and conclusion, finally post limitations of the current study and future research prospects.

## 2 Methodology

### 2.1 Framework

**Figure 1:**
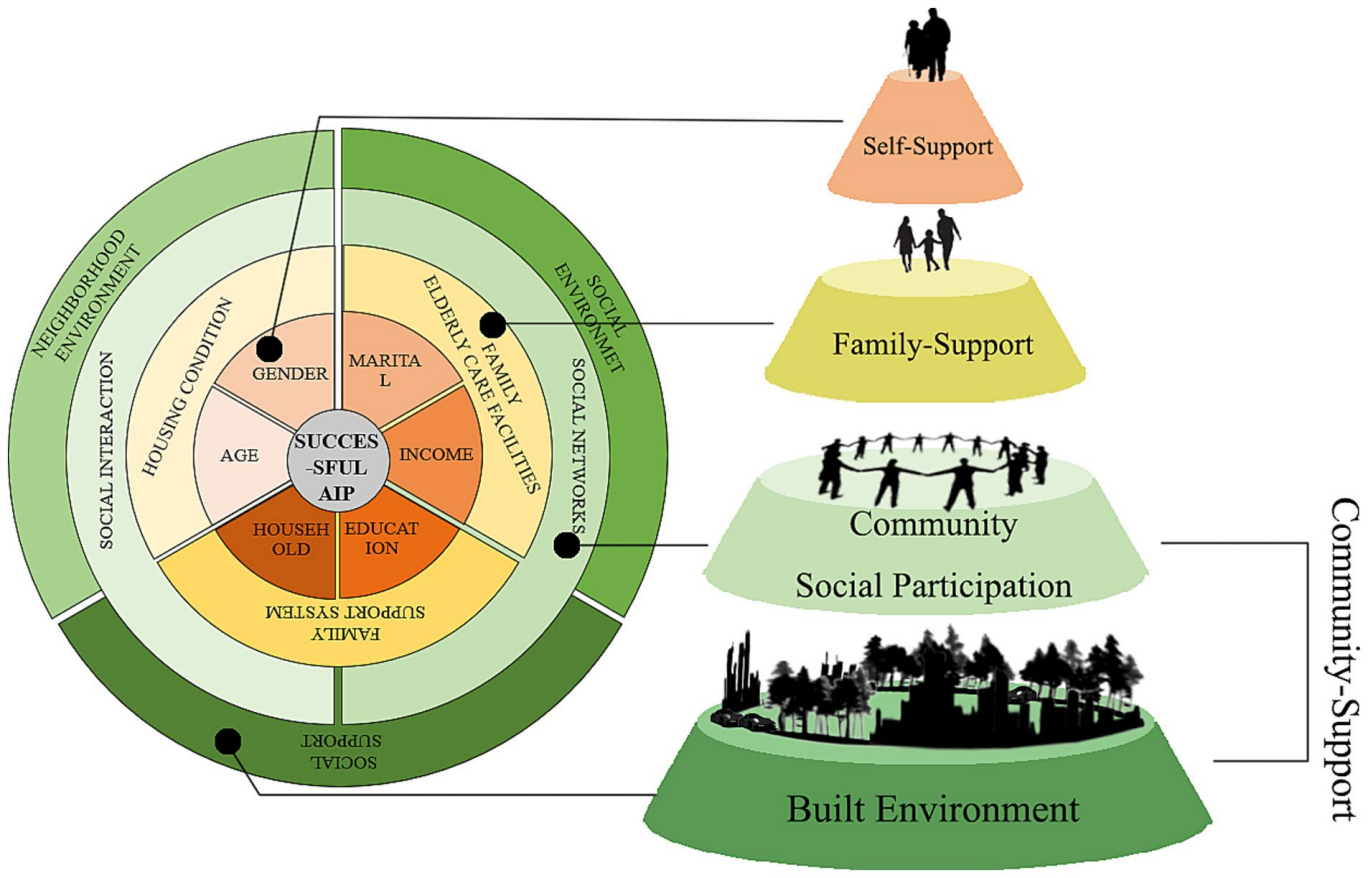
Framework of SAIP.

Based om the P-E (Person-Environment) framework and the socio-ecological theory, we establish an analytical framework showed the built environment at macro, meso, and micro levels collectively influences the SAIP of older adults (Figure 1).

Home-based care, as the micro-level dimension of AIP, represents the most core manifestation of aging within the domestic sphere. It encompasses three indicators: home environment (22), home-based care facilities, and family support systems (12, 53).

Social interaction serves as the primary mode of activity for seniors AIP, constituting their pathway toward proactive health and successful aging. This encompasses two aspects: social networks and participation in activities (1, 25), forming the meso-level dimension of SAIP.

The built environment, as the macro-level external environment for seniors AIP, particularly within the context of neighborhoods with a high concentration of older adults, combines the familiarity of the built environment of the old district with the social ties of acquaintances, fostering a sense of local identity and social well-being among the older adults. Therefore, the external built environment characteristics that support SAIP are jointly constructed through built environment, social environment, and social support system for aging.

### 2.2 Measurement of variables

#### 2.2.1 Measurement of SAIP

The indicators of successful aging encompass self-assessment of health, self-assessment of capabilities, and subjective well-being evaluations (Table 1). Self-assessed health indicators derive from individuals’ reports on their disease conditions, with the total number of chronic disease types used to gage their physical health status. Self-assessment of capabilities is judged by individuals’ self-evaluations of difficulties in their daily living abilities.

**Table 1:** Data sources used for measuring the core indicators.

Indicators	Measurement indicators	M (SD)	Original
Home-based environment			
Living condition	Housing Area	2.770 (1.076)	(38)
Older adults care facilities in home space	Ownership of Home Safety Facilities (Y = 1/N = 0)	0.267 (0.443)	(39)
	Ownership of Health Management Facilities (Y = 1/N = 0)	0.704 (0.457)	
	Ownership of Smart Home Facilities (Y = 1/N = 0)	0.314 (0.465)	(40)
Family support system	Number of Children	1.195 (0.438)	(53)
	Living Relationship with Children (1 = cohabitation/5 = children living in other cities)	3.255 (0.993)	(12, 53);
	Financial Support from Children (1 = never/5 = always)	2.625 (1.101)	(31, 53)
	Visits from Children (1 = never/5 = always)	3.241 (1.063)	(12)
	Online Communication with Children (1 = never/5 = always)	3.902 (0.936)	
Social interaction			
Social networks	Duration of Acquaintance (1 = lower than 6 months/6 = over than 10 years)	5.543 (0.854)	(4, 19)
	Frequency of Interaction with Acquaintance (1 = never/5 = always)	3.941 (1.367)	(19, 41, 42)
Social learning engagement	Frequency of use of older adults learning spaces (1 = never/5 = always)	2.872 (1.475)	
Social activity participation	Frequency of Use of Indoor Leisure Spaces (1 = never/5 = always)	2.872 (1.453)	(43)
	Frequency of Use of Indoor Sports Spaces (1 = never/5 = always)	2.481 (1.460)	(43)
	Frequency of Use of Outdoor Activity Spaces (1 = never/5 = always)	3.919 (1.341)	(43)
Older adults supplies participation	Frequency of Use of Community Health Service Stations (1 = never/5 = always)	3.242(1.084)	
	Frequency of Use of Community Canteens (1 = never/5 = always)	2.207(1.586)	
Built environment			
Macro social environment	Population Density (person/square kilometer)	20406.613 (5098.855)	(43)
	Aging Rate (%)	0.297 (0.027)	
Macro built environment	Floor Area Ratio (FAR) (%)	2.587 (0.423)	(44, 45)
	Building Age	3.012 (0.819)	(46)
Social support system	Accessibility to Green Spaces (≤5-min walk)	0.984 (0.020)	(47)
	Accessibility to Medical Facilities (≤5-min walk)	0.188 (0.165)	(56)
	(≤10-min walk)	0.545 (0.340)	
	Accessibility to Older adults Care Facilities (≤5-min walk)	0.380 (0.326)	(56)
	(≤10-min walk)	0.760 (0.337)	

*The built environment is derived from census data.

Subjective well-being is a crucial outcome of AIP, upon which seniors develop autonomy and environmental initiative (26), becoming the primary factors influencing their mental and social health in later life and necessary for achieving successful and active aging. The measurement of subjective well-being draws on the comprehensive scale developed by Droseltis and Vignoles (27), while also incorporating the willingness to age in place as an indicator of subjective well-being within the framework of SAIP.

The entropy weight method (28) is applied to consolidate these indicators into a dummy variable representing health level, which measures the level of SAIP (Table 2). The greater the degree of dispersion of an indicator, the higher its information entropy, indicating that it provides more information and has a greater impact. Therefore, higher weights are assigned to such indicators, and lower weights are assigned to those with less dispersion.

**Table 2:** Data sources used for measuring the SAIP indicators.

Indicators	Weighted	Original
Health self-assessment	0.04	
X1 Disease	0.04	
Competency self-assessment	0.44	
X2 Basic Self-Care Ability	0.14	(21)
X3 Ability to Conduct Activities at Home	0.10	
X4 Ability to Conduct Activities Outdoors	0.20	
Well-being	0.52	
X5 Respect	0.05	(27, 57)
X6 Convenience	0.05	
X7 Confidence	0.05	
X8 Optimism	0.10	
X9 Familiarity	0.08	
X10 Satisfaction with Life Status	0.09	
X11 Willingness to AIP	0.10	

The loss of mobility capabilities poses the greatest obstacle to SAIP for seniors. The ability to engage in outdoor activities has the strongest impact on it, with an influence coefficient of 0.20, followed by basic self-care abilities, accounting for 0.14 of the total influence. In contrast, the number of diseases has the least impact on it, with a mere 0.04, indicating that in an era of advanced technology and healthcare, chronic diseases among the older adults have gradually mitigated their risks to SAIP within a controllable range.

#### 2.2.2 Measurement of home-based care

As the micro-level manifestation of AIP, home-based care embodies the aging process within the most fundamental domestic space (51, 52), encompassing three key indicators: family environment, family older adult care facilities, and family support systems (Table 1). These indicators include housing area to reflect overcrowding, and the availability of family safety facilities, health management devices, and smart home appliances to gage the older adults-friendliness of household infrastructure. Drawing on the content of home-based care, which typically encompasses economic support, daily care, and emotional comfort (12, 53), a family support system is established.

A strong generational characteristic emerges, with 82.22% of respondents indicating that their children are only-children. Economic support is measured by the frequency with which children provide financial assistance to the older adults, which is not the primary source of income for the majority (56.30% report receiving little or no financial support from their children, once a month or less). Daily care is assessed through living arrangements and the frequency of in-person visits, while emotional comfort is reflected in the frequency of online communication such as phone calls or video chats. Results highlight the prevalence of urban empty-nest seniors, with only 6.91% residing with their children, 41.23% living in different districts, and 11.36% in separate cities. Online communication has become the primary family interaction mode, with 27.65% of seniors engaging in daily phone calls or video chats, compared to 15.56% for in-person visits. While 91.60% of seniors communicate with their children online at least once a week, only 73.83% receive in-person visits.

Housing conditions are generally comfortable, with only 12.10% residing in small apartments under 55 square meters. Most (29.88 and 32.59%) occupy medium-sized apartments ranging from 55 to 89 square meters, indicating a low threat of high-density urban living to residential space. Home health management and monitoring devices are the most common older adults-friendly facilities, with 70.37% of respondents owning equipment such as pulse oximeters, smart blood pressure monitors, glucometers, ECG monitors, and blood cholesterol testers. Conversely, smart devices and home safety equipment are less prevalent, with only 31.36 and 26.67% owning robotic vacuum cleaners, vacuum cleaners, smart locks, smoke detectors, and gas alarms, respectively.

#### 2.2.3 Measurement of social interaction

The meso-level encompasses the interactive social support system for older adults care, with community acquaintance duration and interaction frequency (Table 1) serving as social network indicators, reflecting the strength of community ties. Additionally, the utilization of public spaces is measured to gage community engagement.

Outdoor recreational spaces are frequently used by the older adults, with 48.40% visiting plazas, fitness equipment, and walking paths daily. Community health service stations follow closely, with 40.74 and 22.22% of respondents visiting monthly or weekly for regular consultations and medication refills. The utilization of learning spaces, indoor leisure areas, and activity spaces is more polarized, with 31.36, 27.90, and 40.00% of respondents never using these facilities, while 45.44, 38.27, and 25.67% visit them more than once a week. Community cafeterias have the lowest usage rate, with 57.28% of respondents never utilizing this older adult care facility.

#### 2.2.4 Measurement of built environment

Macro-level indicators focus on the objective context of high-density double-aging neighborhoods (Table 1), encompassing average FAR as indicator of high-density built environments, building age as a measure of aging infrastructure, population density as a marker of high-density living, and aging rate as an indicator of population aging. Accessibility to public supplies such as parks, green spaces, medical facilities, and older adults care facilities serves as a proxy for the social support system, reflecting the provision of community-based supplies relevant to older adults living. Given the concentration of older adults daily activities around their residences, two buffer zones (5-min and 10-min walkable areas, corresponding to 240 m and 500 m radii) are established to analyze the accessibility of social support systems.

### 2.3 Study area

**Figure 2:**
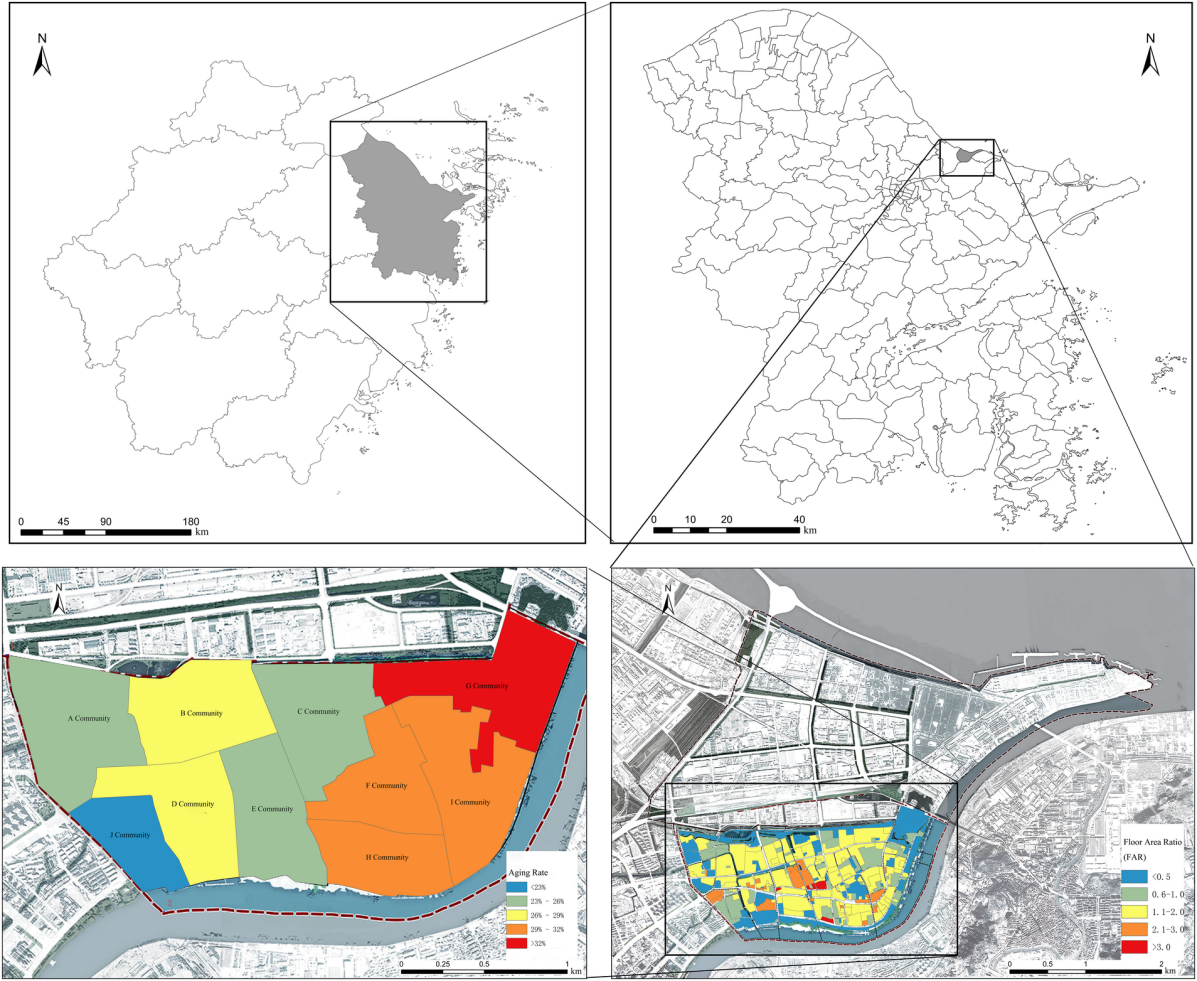
Study area.

ZBS Street is a township street in Zhenhai District, Ningbo City, Zhejiang Province, China (Figure 2), with an administrative area of 21.71 square kilometers. Considering the concentration of the older adults, the urban communities (the old district) within ZBS Subdistrict were selected as the study area.

#### 2.3.1 High density population and high density built environment

The demographic characteristics of ZBS subdistrict exhibit a typical high-density population pattern of an old urban area, with a total population of 73,029, including 48.91% males and 51.09% females, resulting in a gender ratio of 95.7:100, with females slightly outnumbering males. Given that the old town serves as a densely populated residential area, it is characterized by high-density features.

Most residential areas there have a FAR above 1.6 (the State Ministry of Construction requires a minimum floor area ratio of 1.2 for low-rise to high density residential buildings), reflecting a pronounced high-density spatial characteristic. Apart from a few newly built high-rise apartment complexes, a significant number of older residential areas (six stories or fewer) exhibit high-density conditions.

#### 2.3.2 Deep aging and aging neighborhoods

The population structure in study area exhibits a shrinking trend with inadequate vitality. The population pyramid displays a distorted pattern of a narrowing base and widening top. There are 19,326 older adults individuals aged 60 and above, accounting for a 26.46% aging rate. The highest proportion reached 33.73%, with half of the communities having an aging level of over 30%. Among them, 48.25% are male and 51.75% are female, with slightly more older adults females than males. The majority of the older adult population consists of vibrant seniors, with 84.28% aged 60–79, and only 15.72% aged 80 and above. According to the Seventh Population Census, there are 83 unhealthy older adults individuals in ZBS subdistrict, accounting for 6.51% of those over 65, which is lower than the Ningbo city’s average.

ZBS subdistrict is home to numerous aging neighborhoods, mostly constructed in the 1970s and 1980s, housing a large number of long-term residents, including the first generation of urban construction and urbanization populations. Most residential buildings are low-rise apartments of seven stories or fewer, with narrow alleyways, unplanned layouts, and a lack of aging-friendly facilities such as elevators. Moreover, due to structural and layout issues, installing external elevators poses safety risks and spatial challenges. Housing units are predominantly small-sized, with most original housing units measuring under 55 square meters, while some commercial properties reach 80–90 square meters, leading to common overcrowding issues.

### 2.4 Data sources

#### 2.4.1 Field survey

The questionnaire survey targeted older adults households aged 60 and above within the ZBS subdistrict. The questionnaire aimed to collect information on individual socio-economic characteristics and family composition, availability of home-based older adults care facilities, social network connections, participation in community activities, as well as local identity and self-assessed health status. A semi-structured interview was conducted to provide a supplementary analysis of the questionnaire. A total of 500 questionnaires were collected, and after excluding available and incomplete responses, 405 fully qualified questionnaires remained for analysis (Table 3).

**Table 3:** Demographic characteristics of the participants (*N* = 405).

Indicator	Description	Percentage (%)
Gender	Male	41.73
	Female	58.27
Age	60–64	19.01
	65–69	20.25
	70–74	27.9
	75–79	16.3
	80–84	10.12
	85–90	4.44
	Over 90	1.98
Marital status	Unmarried	0.49
	Married	80.49
	Divorced	2.47
	Widowed	16.54
Income (RMB/month)	Less than 1,500	1.98
	1,500–2,490	3.7
	2,491–3,999	21.98
	4,000–5,999	44.2
	6,000–8,477	21.98
	8,478–9,999	3.95
	10,000–14,999	1.98
	Over 15,000	0.25
Education	Primary school and below	21.48
	Junior high school	42.72
	High school (including technical secondary school and technical school)	28.64
	University (including junior college)	6.91
	Graduate student and above	0.25
Household	ZBS Subdistrict	88.89
	Other cubdistrict in Zhenhai District	5.19
	Other districts in Ningbo	2.72
	Outside Ningbo	2.72

The results indicated a reliability coefficient of Cronbach’s alpha = 0.627, slightly below 0.7, while the validity test yielded KMO = 0.815, exceeding 0.6, with a significant *p*-value ≤0.001, indicating good validity. Table 2 presents the personal attributes and socio-economic characteristics of the respondents. The sample comprised 169 males (41.73%) and 236 females (58.27%). Among them, 338 respondents (83.46%) were in the 60–79 age group, similar to the overall proportion of this age group in ZBS subdistrict. Married individuals constituted the majority of respondents (80.49%), while the widowhood rate was 16.54%. The economic level of the respondents was generally moderate, with 44.20% of them earning a monthly income of 4,000–5,999 yuan. Impressed by historical factors, the overall education level was relatively low, with 21.48% of them having a primary school education or below. Native residents were the primary respondents. Overall, the gender ratio with a slightly higher proportion of female respondents is similar to that found in the Seventh Population Census data and household registration records.

#### 2.4.2 Analytical methodology

**Figure 3:**
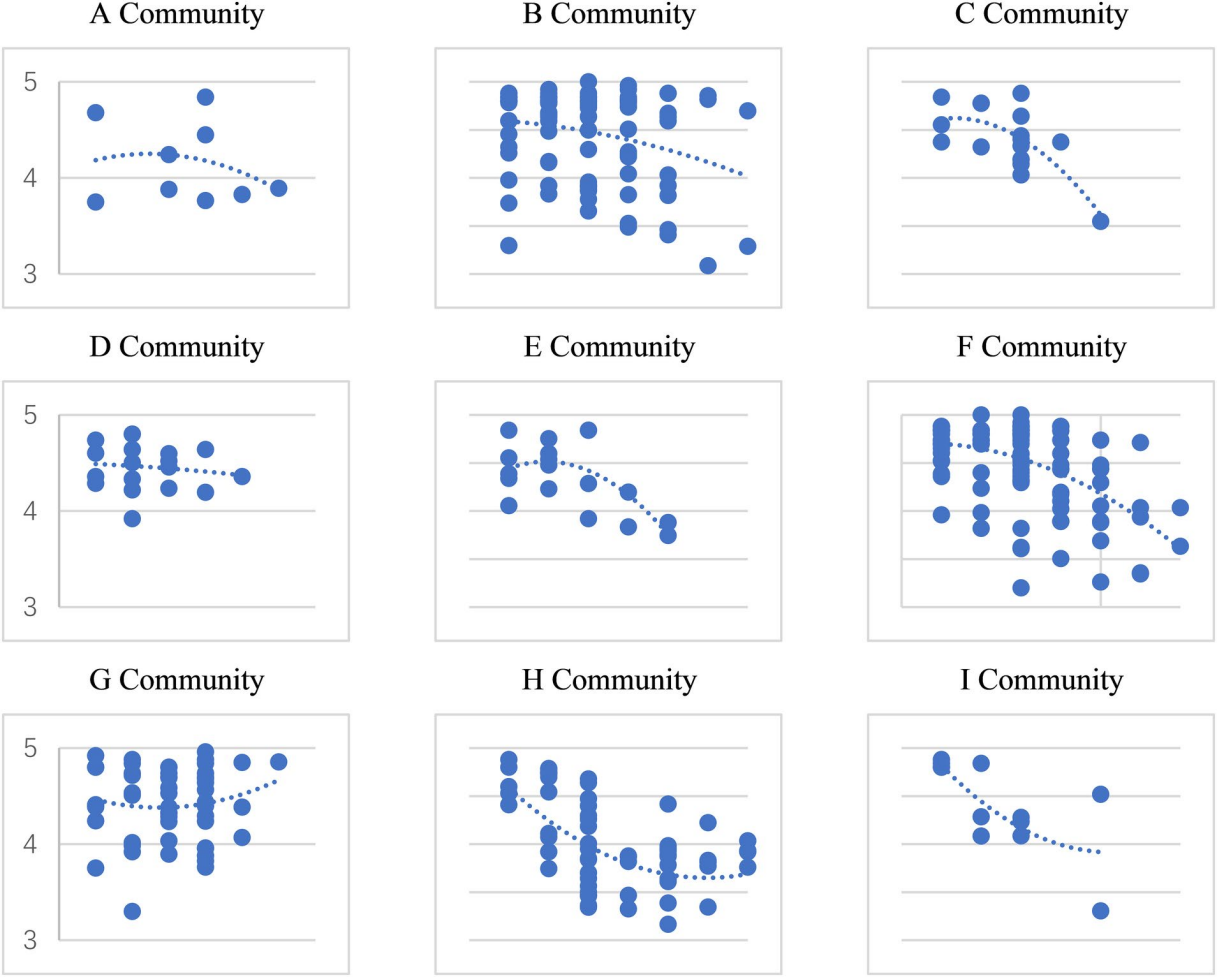
OLS fitting chart of age and SAIP (about **A–I** community in ZBS Street).

The built environment of different communities has a significant impact on the successful aging in place of the older adults (Figure 3). Various influencing factors interact with each other, exerting complex effects on the aging results. To investigate the multi-faceted effects of macro, meso, and micro built environments on self-rated health, a Hierarchical Linear Model (HLM) is employed (Equation 1), controlling for various factors and incorporating random intercepts to capture health variations among individuals. This analytical approach not only adeptly handles the nested structure of the data but also enables the introduction of distinct predictors at different levels of analysis, thereby accommodating more complex models.

The traditional regression analysis is disaggregated into four components using HLM (Table 3). Model 1 encompasses socioeconomic attributes, explaining individual determinants of long-term SAIP. Model 2 extends this by incorporating micro-level household environments, home-based older adults care facilities, and family support systems, elucidating the influence of familial support within the household environment on SAIP. Model 3 builds upon the home-based care foundation by adding built environment conditions, revealing the impact of the built environment on older adults individuals’ home-based care, including macro-level social and built environments, as well as social support systems. Finally, Model 4 integrates social interaction factors, such as social network participation, social learning engagement, participation in social activities, and engagement with older adults care supplies, to assess their contributions to SAIP.


(1) $$Y_{ij}=\beta_{0j}+\beta_{1j}X_{j}+r_{ij}$$

$$\beta_{ij}=\gamma_{00}+\mu_{0j}$$

$$\beta_{1j}=\gamma_{10}+\mu_{ij}$$

## 3 Results

### 3.1 Current home-based care as a necessary condition for SAIP

The relationship between age and SAIP show an inverted U-shaped in Model 1. The age of young-old adults is significantly positively correlated with healthy aging, with a *p*-value of 0.001 for the age group of 60–64 and 0.009 for the age group of 65–69. In contrast, there is a negative relationship in the age of oldest-old adults, including the age groups of 80–85 and 85–89, and those over 90, the intensity of them is shown as −4.088, −5.080 and − 2.969. Among young-old adults aged 60–64, 71.43% are in a state of completely healthy successful aging, but only 12.50% of those over 90 are in such a state (Table 4).

**Table 4:** Results of HLM.

Indicators	Model 1			Model 2			Model 3			Model 4		
	S.D.	T	Sig.	S.D.	T	Sig.	S.D.	T	Sig.	S.D.	T	Sig.
(Constant)		27.514	0.000^***^		16.348	0.000^***^		12.861	0.000^***^		2.411	0.016^*^
Gender	−0.028	−0.596	0.551	−0.029	−0.638	0.524	−0.065	−1.508	0.132	−0.041	−0.975	0.330
Age												
Age of 60–64	0.178	3.216	0.001^***^	0.171	3.179	0.002^**^	0.150	2.930	0.004^**^	0.099	2.011	0.045^*^
Age of 65–69	0.141	2.639	0.009^**^	0.133	2.534	0.012^*^	0.117	2.381	0.018^*^	0.122	2.566	0.011^*^
Age of 74–79	−0.043	−0.819	0.413	−0.043	−0.822	0.411	−0.036	−0.736	0.462	−0.051	−1.075	0.283
Age of 80–85	−0.218	−4.088	0.000^***^	−0.211	−3.773	0.000^***^	−0.172	−3.239	0.001^***^	−0.148	−2.902	0.004^**^
Age of 85–89	−0.257	−5.080	0.000^***^	−0.238	−4.766	0.000^***^	−0.197	−4.163	0.000^***^	−0.153	−3.347	0.001^***^
Age over 90	−0.144	−2.969	0.003^**^	−0.123	−2.500	0.013^*^	−0.081	−1.744	0.082	−0.038	−0.844	0.399
Income	0.058	1.074	0.284	0.051	0.965	0.335	0.025	0.497	0.619	−0.008	−0.169	0.866
Education	−0.066	−1.203	0.230	−0.035	−0.638	0.524	−0.033	−0.637	0.525	0.007	0.132	0.895
Household	0.022	0.474	0.636	0.035	0.766	0.444	0.050	1.166	0.244	0.049	1.195	0.233
Marital Status	−0.002	−0.047	0.963	−0.008	−0.148	0.883	0.012	0.242	0.809	0.029	0.614	0.539
Number of Children				−0.019	−0.371	0.711	−0.025	−0.526	0.599	−0.036	−0.761	0.447
Living Relationship with Children				0.015	0.276	0.783	−0.006	−0.121	0.904	0.004	0.088	0.930
Financial Support from Children				−0.044	−0.812	0.417	−0.024	−0.478	0.633	−0.032	−0.649	0.517
Visits from Children				−0.063	−0.950	0.343	−0.059	−0.937	0.349	−0.040	−0.656	0.512
Online Communication with Children				0.203	4.048	0.000^***^	0.204	4.291	0.000^***^	0.196	4.231	0.000^***^
Ownership of Home Safety Facilities				−0.193	−4.217	0.000^***^	−0.202	−4.644	0.000^***^	−0.155	−3.651	0.000^***^
Ownership of Health Management Facilities				0.150	3.305	0.001^***^	0.093	2.142	0.033^*^	0.095	2.269	0.024^*^
Ownership of Smart Home Facilities				0.063	1.245	0.214	0.075	1.568	0.118	0.091	1.943	0.053
Housing Area (m^2^)												
0–55				−0.035	−0.458	0.647	−0.094	−1.297	0.195	−0.134	−1.878	0.061
55–69				−0.003	−0.036	0.972	−0.163	−1.769	0.078	−0.205	−2.298	0.022^*^
70–89				−0.086	−0.889	0.375	−0.242	−2.617	0.009^**^	−0.225	−2.513	0.012^*^
90–120				−0.067	−0.799	0.425	−0.141	−1.772	0.077	−0.111	−1.452	0.147
Frequency of Use of Older adults Learning Spaces							0.127	2.229	0.026^*^	0.128	2.336	0.020^*^
Frequency of Use of Indoor Leisure Spaces							0.113	1.589	0.113	0.072	1.052	0.293
Frequency of Use of Indoor Sports Spaces							0.028	0.388	0.698	0.033	0.480	0.631
Frequency of Use of Outdoor Activity Spaces							0.180	3.645	0.000^***^	0.206	4.270	0.000^***^
Frequency of Use of Community Health Service Stations							0.007	0.135	0.893	0.021	0.415	0.678
Frequency of Use of Community Canteens							−0.136	−2.263	0.024^*^	−0.152	−2.644	0.009^**^
Duration of Acquaintance							0.174	3.777	0.000^***^	0.127	2.798	0.005^**^
Frequency of Interaction							−0.055	−1.227	0.221	−0.045	−1.030	0.304
Population Density										0.834	2.058	0.040^*^
Aging Rate										0.875	2.095	0.037^*^
FAR										−0.859	−2.578	0.010^*^
Building Age										−0.123	−0.956	0.340
Accessibility to Green Spaces (≤5-min walk)										−0.522	−1.904	0.058
Accessibility to Older adults Care Facilities (≤5-min walk)										−0.958	−1.166	0.244
Accessibility to Medical Facilities (≤5-min walk)										1.862	1.431	0.153
Accessibility to Older adults Care Facilities (≤10-min walk)										0.246	0.948	0.344
Accessibility to Medical Facilities within (≤10-min walk)										−0.619	−0.959	0.338

^∗,∗∗,∗∗∗^p-value denote statistical significance at levels of = 0.05, 0.01, and < 0.001.

Model 2 incorporates the home space and home-based care support into the model. Home-based care mitigates the negative impact of age on SAIP to a certain extent. Compared to Model 1, the T-values of the impact intensity across all age groups in Model 2 have decreased, demonstrating that the traditional family support model for the older adults is indeed an important pathway. Online communication, as a crucial means of emotional comfort, significantly and positively influences SAIP. In contrast, the relationships between living arrangements, financial support, and in-person visits with successful aging are not significant. This indicates that during the rapid process of urbanization, online communication has emerged as a defining characteristic of family support for vibrant urban seniors in the new era.

The possession of home safety devices significantly and negatively impacts the level of SAIP. This indicates that safety monitoring devices, as accident monitoring systems, lack predictive and preemptive functions and do not contribute to promoting successful aging among the older adults. In contrast, the possession of health management devices significantly and positively affects SAIP. By monitoring health in real-time, these devices can promptly identify health issues, serving as important facilitators. Furthermore, under the effect of the built environment and social support, the strength of these effects decreases, suggesting that the community environment effectively assumes part of the responsibility for monitoring, detecting, and responding to health emergencies, thereby promoting SAIP among the older adults.

### 3.2 Social support acts as an important moderator for SAIP

The frequency of usage of older adults learning spaces significantly and positively affects SAIP among seniors with a *p*-value of 0.026. Learning spaces such as universities for the older adults and community lecture halls provide information related to seniors’ daily lives, helping them enhance their personal agency. For instance, knowledge dissemination on disease prevention and treatment can better equip seniors to withstand unknown health risks. Furthermore, interest-based activities, volunteer work, and festive events held in these spaces facilitate the establishment of social networks, enabling seniors to realize their personal values, strengthen their agency, and enhance their self-identity, all of which significantly promote SAIP. The frequency of using outdoor activity spaces also has a significant positive effect. Outdoor activities are effective among seniors. Seniors express a strong demand for open spaces (e.g., plazas, walking paths) that facilitate exercises like square dancing and fitness routines. However, the use of community cafeterias exhibits a significant negative impact. Self-reliance is a crucial source of satisfaction, happiness, and achievement among seniors. At this nascent stage of community cafeterias, their usage is often a forced choice due to limited mobility, failing to actively contribute to SAIP.

The duration of acquaintance with familiar community members significantly and positively effects SAIP. This suggests that in a society of acquaintances, seniors can form stable social networks, engage in activities, and provide mutual support based on geographical proximity and shared interests within old neighborhoods.

The increase in social participation indicators effectively reduces the influence of age on SAIP. Compared to Model 1, the impact of age is reduced to varying degrees for seniors under 90, and the impact disappears for those over 90. Additionally, active community participation effectively effects the effectiveness of home-based care for the older adults. The intensity of online communication among family members increases from 4.048 to 4.291. The impact strength of owning health management devices decreases, while the negative impact intensity of home safety devices increases.

### 3.3 The built environment serves as a significant mediator for SAIP

The demographic homogeneity and high-density characteristics of double-aging neighborhoods provide a more like-minded social and humanistic environment for the older adults to AIP. The high population density in the old city shows a significant positive correlation with SAIP, indicating that a high-density population is conducive to older adults obtaining social support for AIP. Meanwhile, the aging of the population in double-older adults communities also significantly and positively correlates with SAIP. The homogeneity and high population density in double-older adult communities provide a more like-minded social and cultural environment for older adults’ AIP, which has advantages in this regard. Under the influence of the built environment in double-older adult communities, the positive affect intensity of online communication among family members and the duration of acquaintanceship in the community decreases compared to Model 3. At the same time, the influence intensity of family health management equipment and home security equipment ownership decreases compared to Model 2. A strong sense of familiarity and security in the built environment effectively reduces the dependence on acquaintances, and the local identity of the old city brings a positive impact on SAIP.

FAR has a significant negative impact on SAIP, indicating that the congestion caused by high-density buildings in the old city is not conducive to AIP. Notably, the negative impact intensity of small- and medium-sized housing on SAIP increases. Housing with an area of 55–69 square meters has a significant negative impact; the negative impact intensity of housing with an area of 70–89 square meters decreases from 2.617 in Model 3 to 2.513.

The impress intensity of accessibility to green spaces, medical spaces, and older adults care spaces is not significant, indicating that in the highly saturated old city with public facilities, older adults do not perceive a strong distance to access public service resources. However, the frequency of using older adults learning spaces, outdoor activity spaces, and community canteens has an enhanced impact on SAIP, with T-values increasing from 2.229 to 2.336, 3.645 to 4.270, and 2.263 to 2.644, respectively.

The built environment further reduces the influence of age on SAIP. The significant positive effect of the age of young-old adult further decreases to 2.011. The significant negative influence intensity of the age of older-old adults (80–85 years old and 85–89 years old) effectively decreases to 2.902 and 3.347, respectively. Notably, the significant positive affect intensity of 65–69 years old is 2.566, slightly higher than 2.534 in Model 2 and 2.381 in Model 3 but lower than 2.639 in Model 1. Older adults in this age group are at a crucial stage of whether they can achieve SAIP and are more sensitive to the influence of the built environment. In the process of old city renovation and the construction of age-friendly communities, this group may become important evaluators.

## 4 Discussion

### 4.1 Symbolic characteristics of home space and socioeconomic status jointly promote SAIP

Driven by the rapid urbanization and the profound effect of Confucianism, the traditional Chinese filial piety culture, which believes that “lies in the substance rather than the appearance,” has taken on a new look in a new era. The home space is not only the physical location and primary living place for the older adults, but also an imagined and metaphorical space for their emotions and sense of belonging (54). Therefore, family support for aging still plays an indispensable role, consistent with the conclusions of existing studies (29, 30). At the same time, the situation of empty-nesters living alone has no longer been the main cause of deprivation in AIP, and family spiritual comfort represented by intergenerational communication has become an important source of inequality in AIP. We also found that in families with vibrant seniors who possess certain socioeconomic conditions, family support for aging has, to a certain extent, transcended traditional economic (31) and caregiving needs (12). Communication and interaction through various channels have become important sources of spiritual comfort for the older adults, further revealing the spiritual essence of filial piety culture.

SAIP is not significantly affected by socioeconomics. Under the highly covered urban pension insurance system, most older adults individuals’ economic conditions can already support their basic needs for retirement. This finding differs from Lum et al. (32) and Andrew Scharlach’s (33) conclusion that the “stuck in place” phenomenon among the older adults is constrained by economic and social resources among low-income older adults Chinese city. On the one hand, this indicates that seniors actively choose to age in place rather than being “stuck in place.” Their familiarity and identification with the environment, attachment to the home space, and protection of the home culture, as well as their preference for a free and independent lifestyle, are the core reasons for their preference to age in place. On the other hand, it also suggests that despite robust financial support systems for retirement, other conditions are still necessary for achieving SAIP.

### 4.2 Familiarity and identity with the built environment as essential components of SAIP

The built environment serves as the primary public space for the older adults to AIP, and it is the core pathway to fulfill their social and psychological needs that cannot be fully met by home space, such as a sense of belonging, socialization, independence, autonomy, and good relationships with the vicinity. These needs are manifested through social participation and social interaction. Contrary to Ahmed et al. (19) that there is no correlation between social participation and the preference for AIP, we found that active participation in community learning and outdoor activities can effectively promote SAIP. Such community participation can help older adults better integrate into the local community, establish healthy social networks, and make a sense of place identity. Especially in the familiar place and space of old district where people know each other well, community participation and acquaintance network become crucial channels for social interaction. As indicated in existing research that emphasizes social experiences across different spatial scales, the closeness of the older adult’s connection to their place of residence and their sense of place identity can enhance their environmental agency (55). We further discovered that this ability to engage can effectively improve the level of successful AIP, better fulfilling their independent and autonomous well-being needs. In these acquaintance-based old district, the more permanent residents are, the more likely they are to achieve positive outcomes of SAIP.

This study finds that high density population is conducive to AIP, and an aging community also promotes SAIP. This echoes the findings of Fitzgerald and Caro (34), who concluded that population density is one of the prerequisites for making a community senior-friendly. However, the high density of old cities represented by high FAR has a significant negative impact on SAIP. While some existing studies suggest that double-aging neighborhoods face issues in livability and resilience (4), making them unfavorable for AIP, our findings share some similarities with Ewen (35) that old communities have limitations in the service targets, accessibility, practicality, and scale allocation of public spaces. Fitzgerald and Caro (34) argues that the diversity and concentration of the older adults population require corresponding supportive living environments, and suggest that enhancing community service accessibility (35) and the number of community support networks (36) can effectively promote successful aging in place. Building upon this foundation, we have discovered the unique characteristics of the built environment in old cities and their distinct impacts on SAIP. A certain amount of excess healthcare resources that we have derived from previous study is an effective way to improve the dual structure of urban and rural areas and narrow the urban–rural gap in the process of rapid urbanization, and it can effectively alleviate the problem of deprivation of healthcare resources for vulnerable groups such as the older adults (28). We further validate this spatial characterization of the high-density distribution of public facilities of old cities in this study, which is shown as public service facilities are relatively saturated. However, the high-density distribution of these facilities fails to further promote SAIP because of the potential and specific ways, methods, and frequency of utilization. Therefore, in the renovation of double-aging neighborhoods, differentiating from the functional integration in traditional urban renewal, in-situ updating of fragmented functional facilities and preservation of interpersonal relationships can better support the AIP of older adult residents. Preserving the “vibrancy” of old district is a crucial goal in their renovation. Under the statuses of maintaining high accessibility, alleviating the environmental exposure of crowding and noise in high-density spaces to a certain extent is an effective path toward transitioning to age-friendly communities.

### 4.3 A smart transition path from a double-aging neighborhoods to future community

The unique multi-agent nature of AIP distinguishes it from traditional home-based and institutional aging, requiring collaboration among local governments, market institutions, social organizations, and family members (10). This paper analyzes the age groups of local older adult people and their levels of SAIP, contributing to a nuanced understanding of the AIP needs of different age groups. The study finds that the local environment effectively facilitates the SAIP of the younger older adults group, who have strong social participation needs and community mobility. Thus, this group can be a priority for future community construction and the development of smart AIP in double-aging neighborhoods. In contrast, the older adults group aged over 80, due to significant declines in their physical health and mobility, are compelled to reduce their local activities. As a result, the convenience and quality of community-based care become increasingly important. Isolated by the built environment of their communities, so this group should be the potential focus and target.

In double-aging neighborhoods, focusing on the sense of gain, happiness, and security of the older adults and fostering age-friendly communities represent essential elements of future community development. Smart aging is a hot topic in today’s era. Ajani and Olapade (37) indicate that retrofitting and smart house technology can be utilized to transform unsuitable home environment into age-friendly spaces, and empirical studies that examine the impact of retrofitting and smart home technology on aging-in-place are necessary in the future. Therefore, our study found that household smart healthcare devices are widely used and support SAIP. The research validates that the use of these health management and monitoring devices can effectively help seniors understand their health status, promote SAIP, and provide strong evidence for smart aging initiatives. It is noteworthy that regarding community-based care, the study revealed a significant negative correlation between the utilization of community canteens and SAIP. In the process of functional renewal and digital construction in old districts, it is an effective policy support and an important path for the construction of age-friendly communities and realize the successful aging, to popularize the supply of welfare, such as community older adults-care facilities and digital older adults-care facilities on a wider scale, help local older adults bridge the digital divide, and better protect the well-being of the older adults.

## 5 Conclusion

Based on a survey of SAIP among the older adults population in ZBS Street, this study employed a series of HLM models to analyze the multiple-scale built environment on the self-assessed health status of the older adults. The results show that: (1) Age is the most critical individual attribute factor affecting SAIP, exhibiting an inverted U-shaped relationship. Especially, the results of SAIP among the older adults aged 65–69 are sensitive to the feedback of the built environment; (2) Based on a certain economic foundation, older adults people have more demands of spiritual comfort, and the symbolic characteristics of home space jointly promote SAIP; (3) Acquaintance-based society and community participation spaces have a positive impact on SAIP, which replace the role of home-based care in influencing SAIP; (4) The high-density built environment effectively solves the problem of spatial deprivation which is developed form the differences in accessibility of public resources, and the proper utilizes of these facilities becomes an important factor affecting SAIP; (5) The sense of local identity in double-aging neighborhoods, formed by long-term residence, effectively helps the local older adults to age successfully in the local context.

The impact of the community-built environment on health is a complex and promising research topic, especially when neighborhood spaces become the primary activity venues for the older adults, making the built community environment a crucial factor influencing the older adult health and AIP. It is demonstrated that the social environment can effectively compensate for the weakening of home-based older adults care support, validating the effectiveness of the local older adults care model in the context of China’s rapidly aging society. And it shows that the environment of dual-older adults communities effectively promotes SAIP among the older adults, indicating the need to re-examine the old characteristics in the process of old city renovation. By leveraging the cultural heritage and sense of place attachment behind these old characteristics, we can build age-friendly communities without the need for complete urban redevelopment. This provides a new path for the creation of age-friendly communities.

However, while the multi-level analysis perspective offers insights into the influence intensity of different factors on SAIP, there are limitations, and further exploration of the mechanisms through which multi-scale built environment impact SAIP is warranted. How to better meet the needs of the older adults for social care and ensure their well-being in the future community is an important proposition for the construction of age-friendly communities in China. Moreover, current research findings are limited to urban areas with robust economic and welfare systems, necessitating further studies in rural regions with weaker socioeconomic conditions.

## Data Availability

The datasets presented in this article are not readily available because the original contributions presented in the study are included in the article/supplementary material, further inquiries can be directed to the corresponding authors. Requests to access the datasets should be directed to Yue Qian, qiany_qy@163.com.
